# Supplementary material for: An inhalable nanoparticulate STING agonist synergizes with radiotherapy to confer long-term control of lung metastases
Source: Nat Commun. 2019 Nov 8;10:5108. doi: 10.1038/s41467-019-13094-5 (PMC6841721; doi:10.1038/s41467-019-13094-5)
Supplement: Supplementary file 1 — Supplementary Information [file 41467_2019_13094_MOESM1_ESM.pdf]

Supplementary Information

An inhalable nanoparticulate STING agonist synergizes with radiotherapy to confer long-term control of lung metastases

Liu et al.

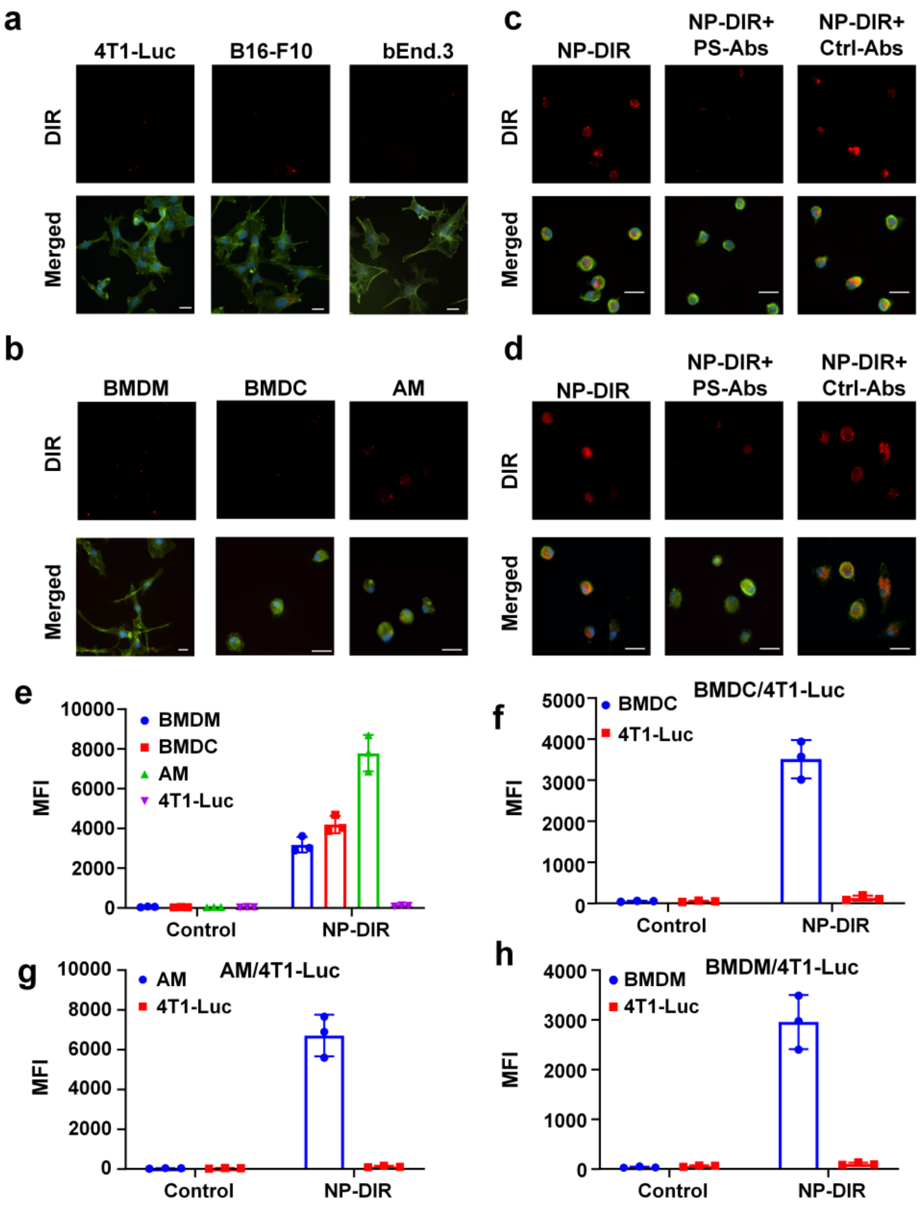

Supplementary Fig. 1. In vitro specific uptake of PS-NP by APCs is mediated by surface exposed PS. a, Minimal uptake was observed after 30 min incubation of the DiR labeled PS-NP (red) with 4T1-luc or B16-F10 cancer cells or mouse vascular endothelial bEnd.3 cells. **b**, Minimal uptake of the anionic phosphatidic acid (PA)-coated NP by BMDM, BMDC and AM after 30 min incubation. **c and d,** PS-NPs were pretreated with/without anti-PS antibody for 2h and then incubated with alveolar macrophages (**c**) or BMDC (**d**) for 30 mins. Fewer DiR signals were detected in the APCs incubated with the anti-PS antibody-treated PS-NPs, while massive uptake

observed for the PS-NPs treated with an irrelevant control antibody (Ctrl Ab). Cytoskeleton (Phalloidin, green), Dapi (blue) for nuclei staining. Similar results observed in n=3 biologically independent experiments. Scale bar=20 μm. **e-h,** Flow cytometry analysis of specific uptake of NP-DiR by APC cells. Incubation of APC cells or 4T1 cancer cells with NP-DiR (**e**) or the co-cultured APC cells with 4T1 cells further incubated with NP-DiR for 30 mins (**f-h**). Data shown from n=3 biologically independent experiments. Source data are provided as a Source Data file.

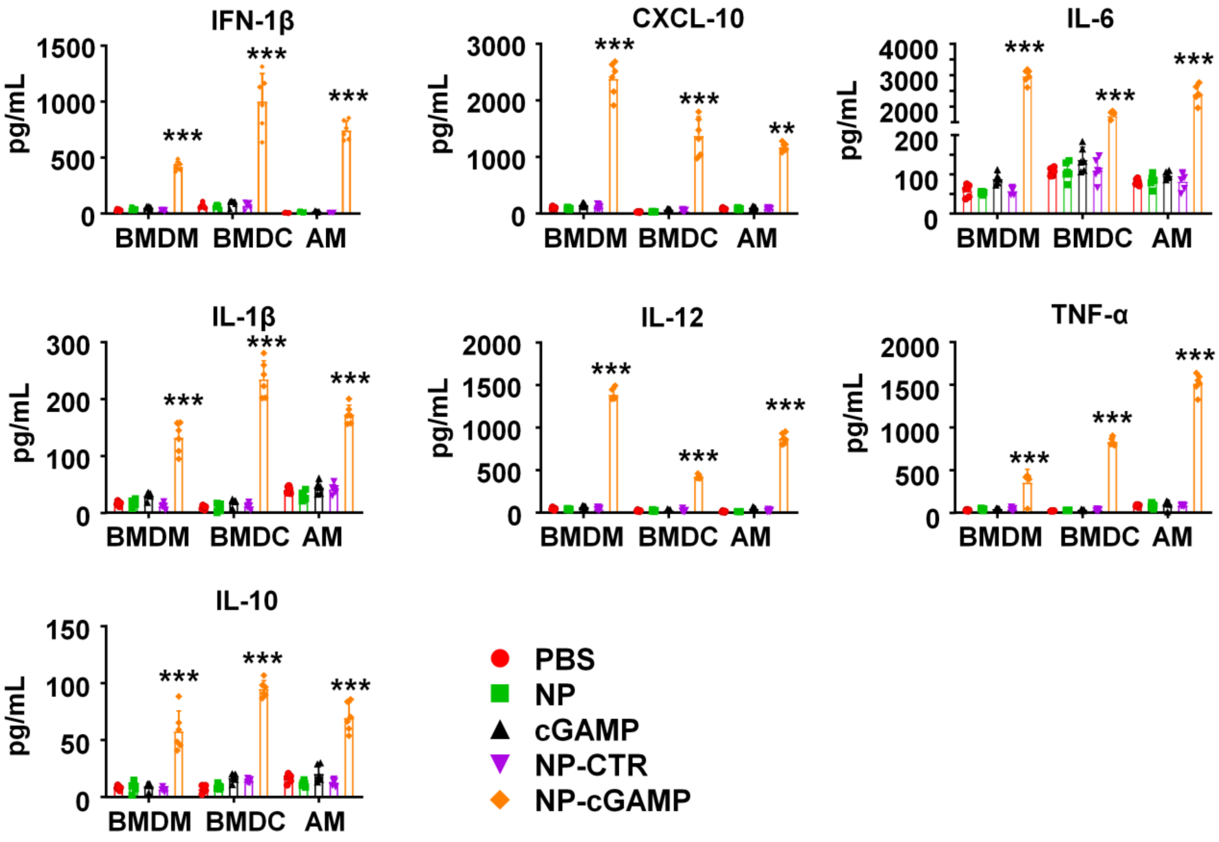

Supplementary Fig. 2. NP-cGAMP induces APCs to produce type I IFN and other cytokines. Cell culture medium from BMDM, BMDC, and AM after indicated treatment for 8h was analyzed by ELISA. Data shown as mean ±SD of 6 biologically independent experiments. * $p<0.05$; ** $p<0.01$; *** $p<0.001$ by Student's T-test. Source data are provided as a Source Data file.

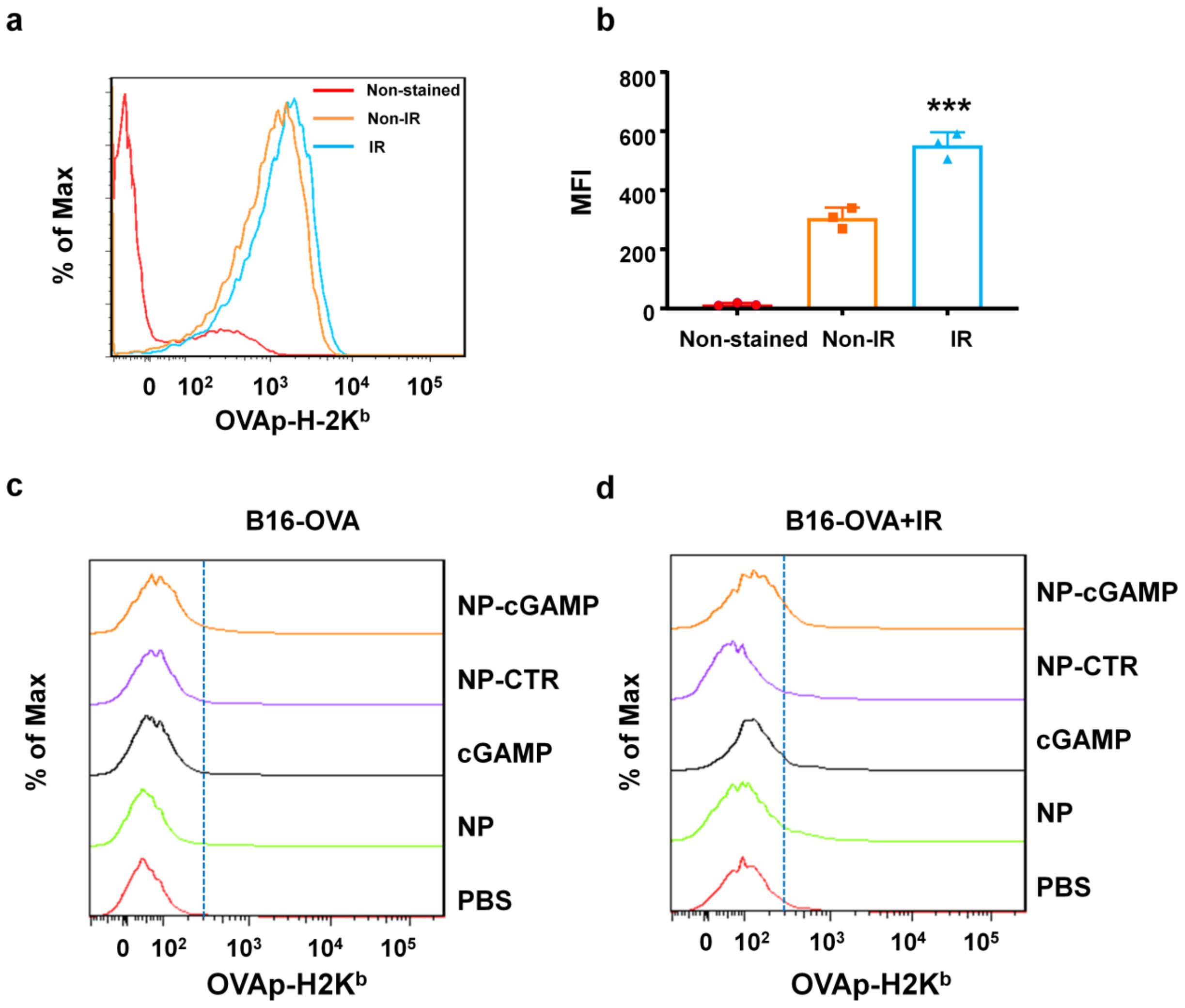

Supplementary Fig. 3. Radiation induces exposure of tumor antigen. a, B16-OVA cells treated with/without a single dose of 20 Gy IR were continued to culture for 72 h and then stained with anti-H2Kb bound to SIINFEKL antibody. A right shift of the IR curve and a significant increase in MFI of the irradiated cells (**b**) was observed by FACS. Representative OVAp-H2Kb expression in BMDCs after cultured with the non-irradiated (c) or irradiated (d) B16-OVA cells. Similar results observed in n=3 biologically independent experiments. *** p<0.001 by Student's T-test. Source data are provided as a Source Data file.

a

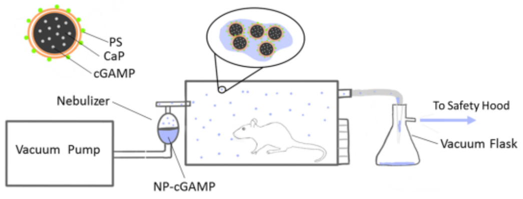

b

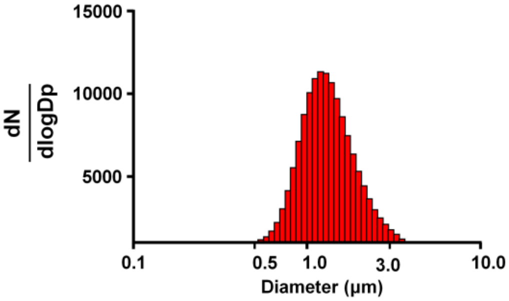

c

RhoB Concentration in Reservoir (μg/mL)	Inhalation Time (min)	HPLC determined RhoB Concentration (ng/g tissue)	Retention rate in Lung
16.0	28.0	264.2±56.1	25.0±3.4%
32.0	28.0	501.4±47.2	22.8±4.9%
16.0	56.0	543.7±62.0	26.4±3.1%
32.0	56.0	976.9±87.6	26.8±2.6%
			Mean: 25.3±2.3%

d

$$M_{drug} = C_{resreservoir} \times C_{Aerosol} \times V_{respiratory} \times RR \times T$$

e

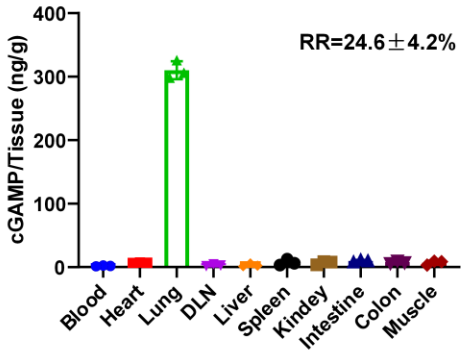

Supplementary Fig. 4. Preparation, characterization and dose determination of aerosolized NP-cGAMP. a, Scheme of nebulized aerosols containing PS-coated NP-cGAMP inhaled by mice. NP-cGAMP was aerosolized by a vacuum pump supplying pressure to a medical nebulizer, and was delivered to a custom-fabricated airtight chamber, where droplets were inhaled by mice. **b**, Representative number–size distribution (dN/dlogDp, cm^{-3}) of aerosols from n=3 biologically independent experiments (mean aerodynamic diameter = 1.38 μm and a geometric SD = 1.25). **c**, Determination of cGAMP dose delivered to lung after inhalation was based on quantitative HPLC measurements of NP-Rhod-b in lung metastases-bearing lung tissues in mice (n = 3/group). HPLC analysis was conducted by applying various concentrations or durations of inhaled NP-Rhod-b. **d**, The mathematical model used to estimate the dose of aerosolized drugs deposited to lung. M_{drug}, drug dose in lung (ng); $C_{reservoir}$, Drug concentration in reservoir (ng/ml) ; $C_{aerosol}$, Aerosol concentration (ml/l) ; $V_{respiratory}$, mice

respiratory volume rate (ml/min); RR, Retention Rate (%) ; T, Inhalation time (min). **e**, HPLC measurements of cGAMP concentrations in various tissues and organs after lung metastases-bearing mice inhaled PS-NP encapsulating cGAMP-FITC. Source data are provided as a Source Data file.

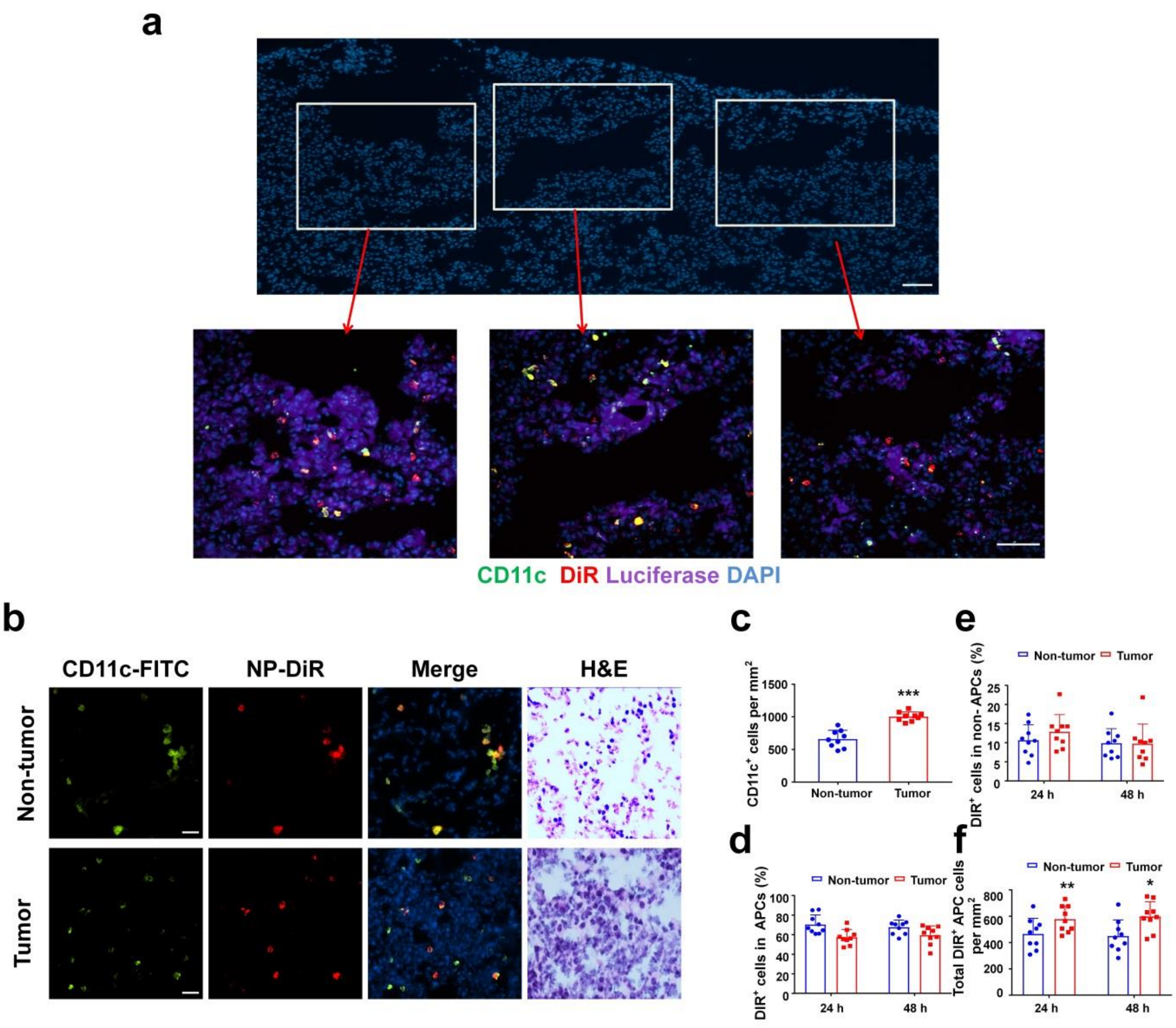

Supplementary Fig. 5. Immunohistochemistry of APC-targeted delivery of PS-NP in lung metastases. The 4T1 lung metastases-bearing mice (n =3 biologically independent mice) were sacrificed at 24h or 48h after inhalation of NP-DiR. Frozen sections of lung tissues were immunostained with anti-CD11c antibody (green, APCs), anti-luciferase (purple, tumor cells) and correlated with DiR signals (red, NP-DiR) and counterstained with Dapi (Blue). **a**, Individual regions were selected from a larger area of tumor-bearing lung tissues 24 h post inhalation. Merged images revealed DiR signals overlapping with CD11+ APCs. Scale bar= 50 μm. **b**, Representative 24h images of a region of the non-tumoral lung tissues (top) and a lung metastasis (bottom). Scale bar = 20 μm. **c**, Counting of CD11c+ APCs showed significantly more APCs in the lung metastases than non-tumoral lung tissues (*** $p<0.001$; by Student's T-test). **d**, NP-DiRs were found to co-localize with 57% and

70% of APCs in tumor and non-tumoral tissues, respectively, which sustained at a high level (60% vs 67%) at 48h. **e**, Only a small fraction of NP-DiRs was seen outside APCs. **f**, There were significantly more NP-DiR-captured APCs in lung metastases than normal lung (* p<0.05, ** p<0.01; by Student's T-test). Data shown as mean ±SD of n=9 biologically independent samples. Source data are provided as a Source Data file.

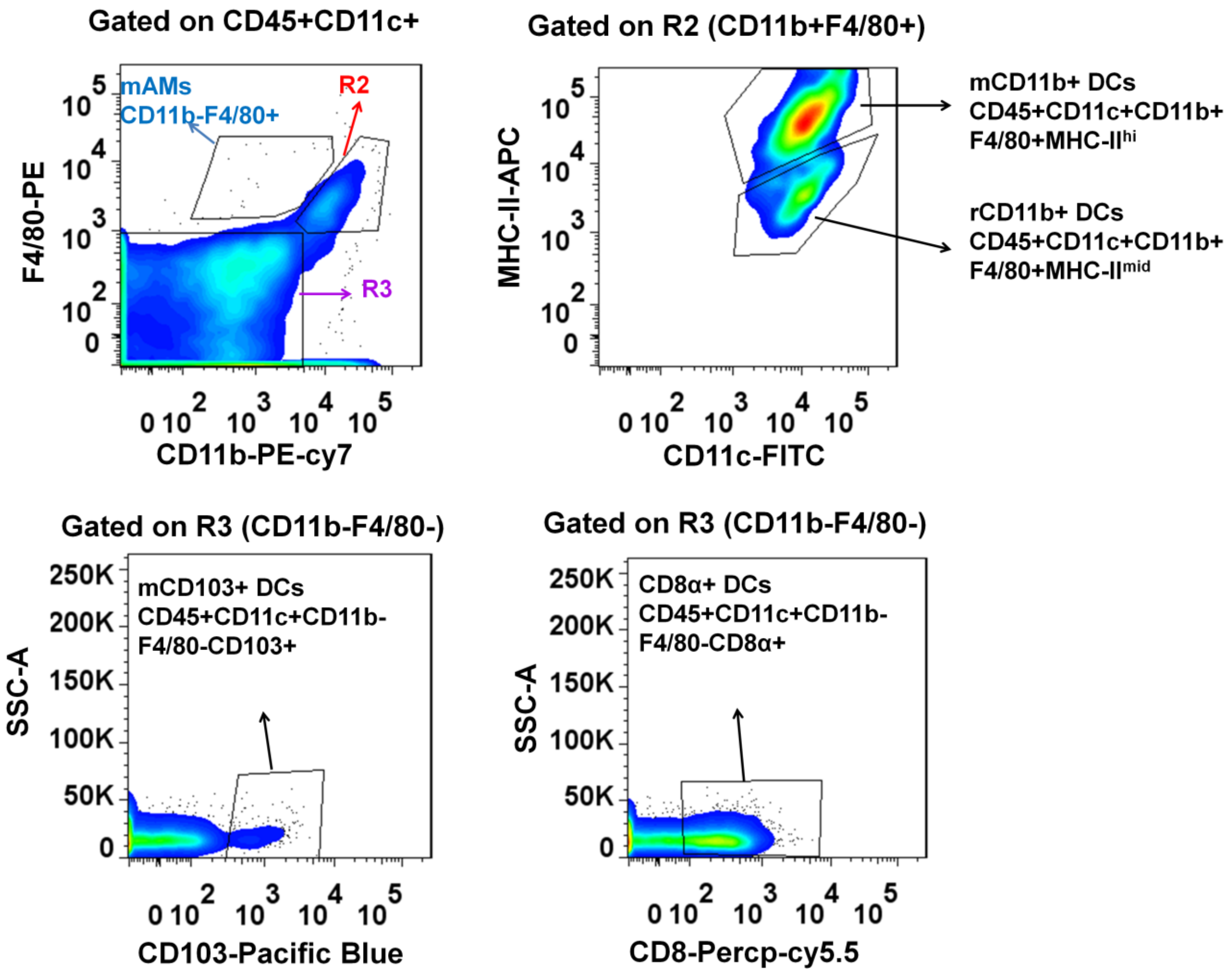

Supplementary Fig. 6. FACS gating strategies for stratification of APC subsets in TDLNs.

Non-DLN resident mCD11b^{+} DC: CD45^{+}CD11c^{+}CD11b^{+}F4/80^{+} MHC-II^{+};

DLN resident rCD11b^{+} DC: CD45^{+}CD11c^{+}CD11b^{+}F4/80^{+} MHC-II^{mid};

CD103^{+} DC: CD45^{+}CD11c^{+}CD11b^{-}F4/80^{-}CD103^{+};

CD8α^{+} DC: CD45^{+}CD11c^{+}CD11b^{-}F4/80^{-}CD8α^{+}

The above gating strategy was used to generate data in Fig. 3g.

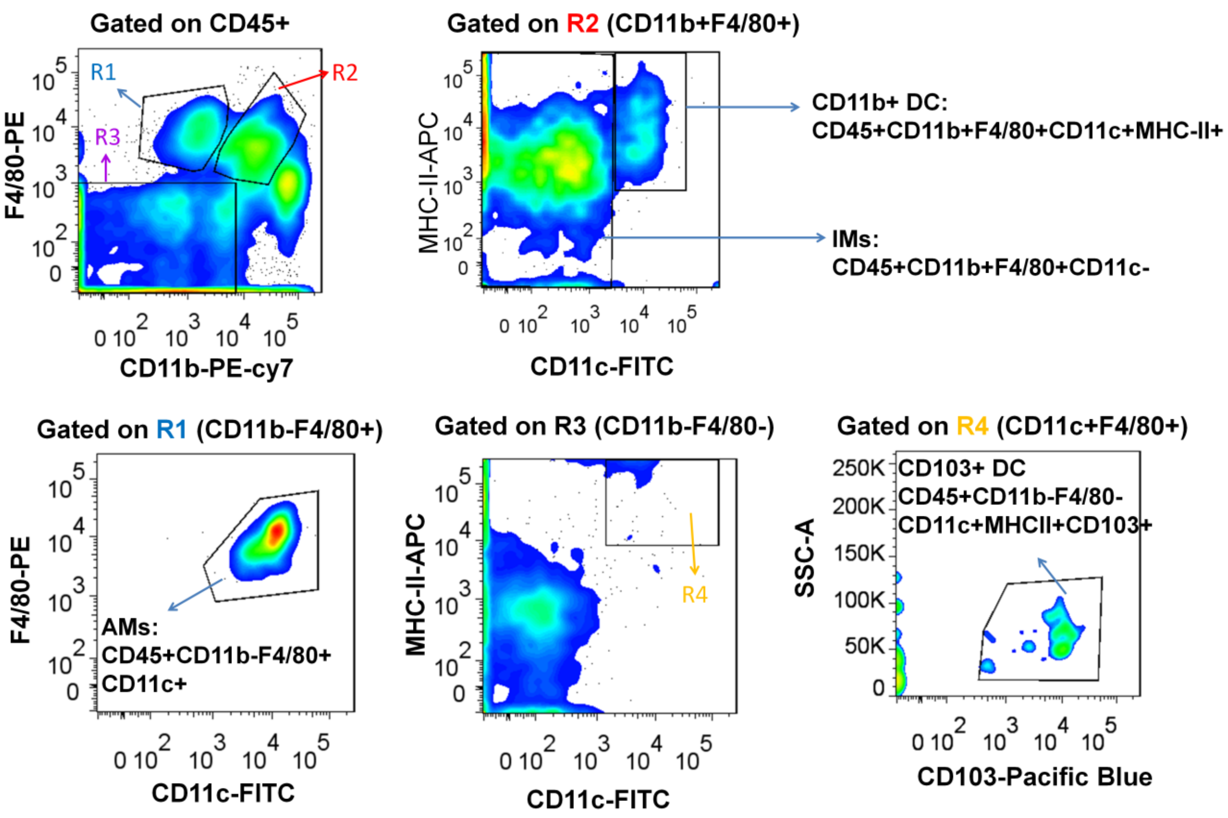

Supplementary Fig. 7. FACS gating strategies for stratification of APC subsets in lung metastases.

AM: $CD45^{+}CD11b^{-}F4/80^{+}CD11c^{+}$;

$CD11b^{+}$ DC: $CD45^{+}CD11b^{+}F4/80^{+}CD11c^{+}MHC\text{-}II^{+}$;

$CD103^{+}$ DC: $CD45^{+}CD11b^{-}F4/80^{-}CD11c^{+}MHC\text{-}II^{+}$ $CD103^{+}$;

IM: $CD45^{+}CD11b^{+}F4/80^{+}CD11c^{-}MHC\text{-}II^{-}$

The above gating strategy was used to generate data in Fig. 4c,d and Supplementary Fig. 9.

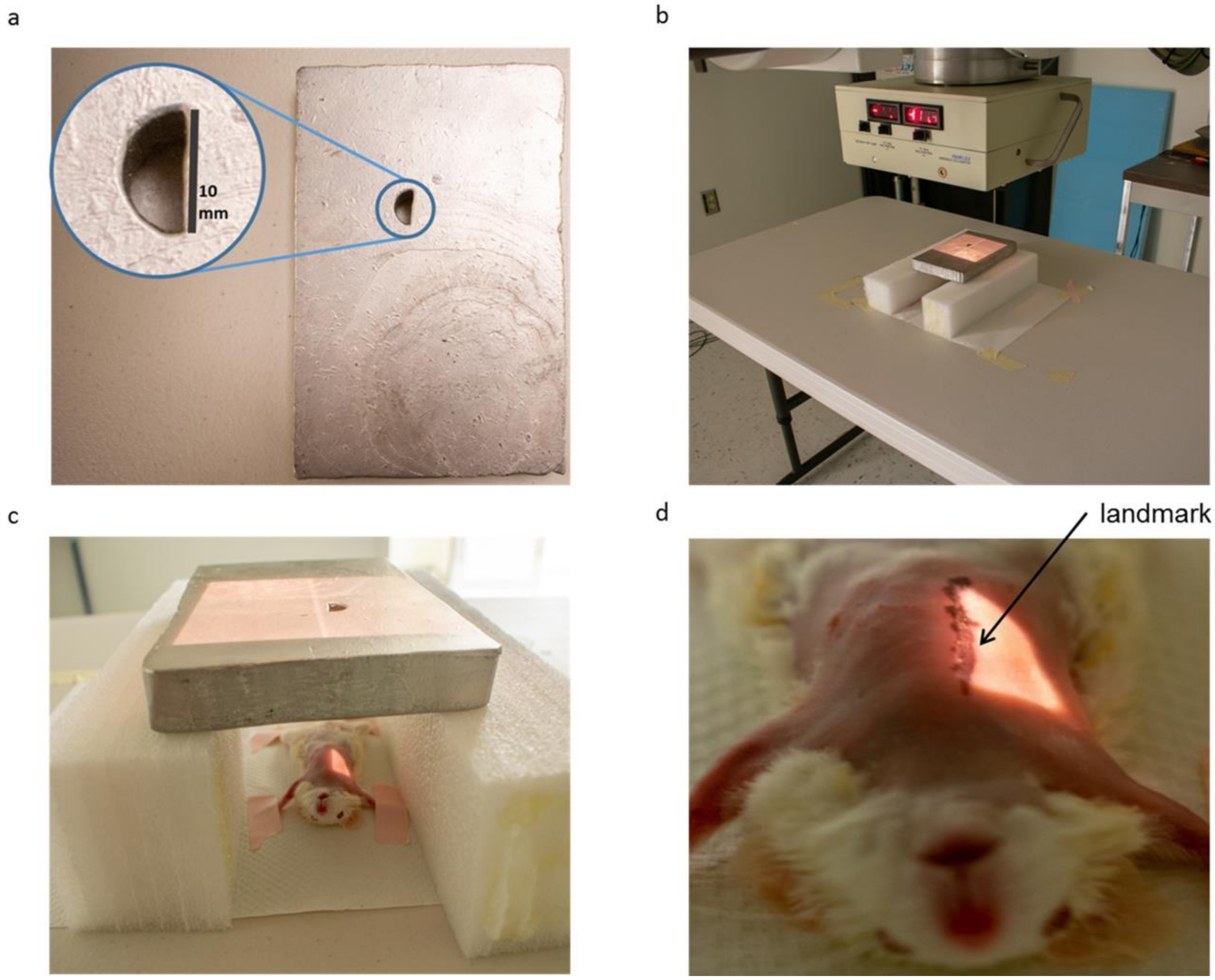

Supplementary Fig. 8. Irradiation setting for targeted right lung radiation. a, The custom-made alloy shied with a half circle of 10 mm diameter. **b-d,** The IR setting with an anesthetized animal and the projected X-ray radiation coverage (yellow light field). The landmark (arrow) drawn at the initial fraction was used to ensure the reproducibility of the IR for the following 2nd and 3rd fraction.

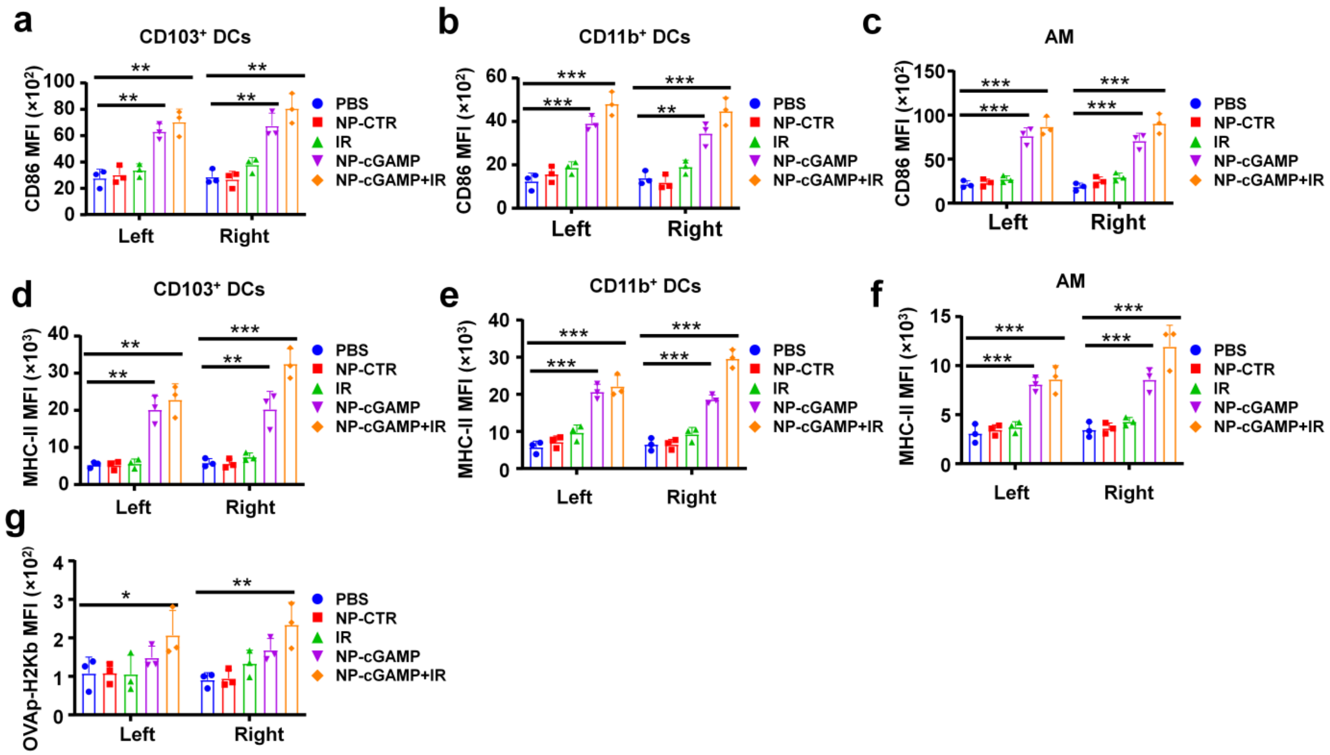

Supplementary Fig. 9. In vivo activation of APCs in B16-OVA lung metastases by NP-cGAMP inhalation. 24h after the last inhalation, the mice under indicated treatment were sacrificed and both metastases-bearing lungs were dissected for FACS analysis of the co-stimulatory molecule, CD86 on CD103+DCs (**a**), DC11b+DCs (**b**) and AM (**c**). **d-f**, Similarly, MHC-II expressions on DCs and AMs were quantified. **g**, Expression of SIINFEKL-MHC-I complex on CD11b+DCs. Data shown as mean ±SD of n=3 biologically independent mice/group. * $p<0.05$, ** $p<0.01$; *** $p<0.001$ by Student's T-test. Source data are provided as a Source Data file.

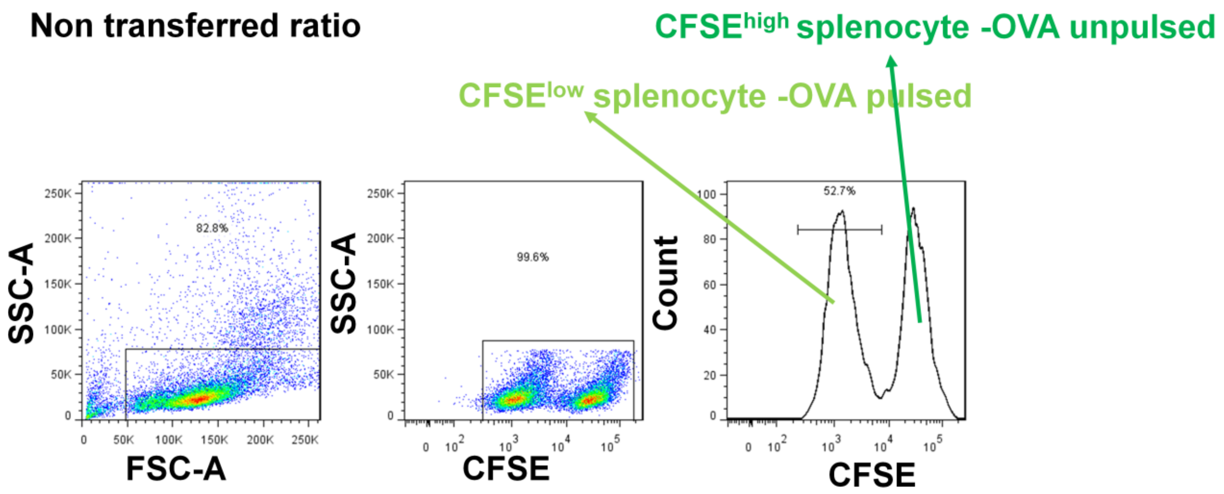

Supplementary Fig. 10. Preparation of OVA-splenocytes for in vivo VITAL assay. Spleen cells from naïve C57BL/6 mice were isolated and half of the cells were pulsed with $OVA_{257\text{-}264}$ for 2h in complete medium. The non-pulsed and OVA-pulsed cells were labelled with high (0.5) or low (0.05) CFSE, respectively. The cells were equally mixed before injection to mice. The cell mixture shown was used as non-transferred control ratio.

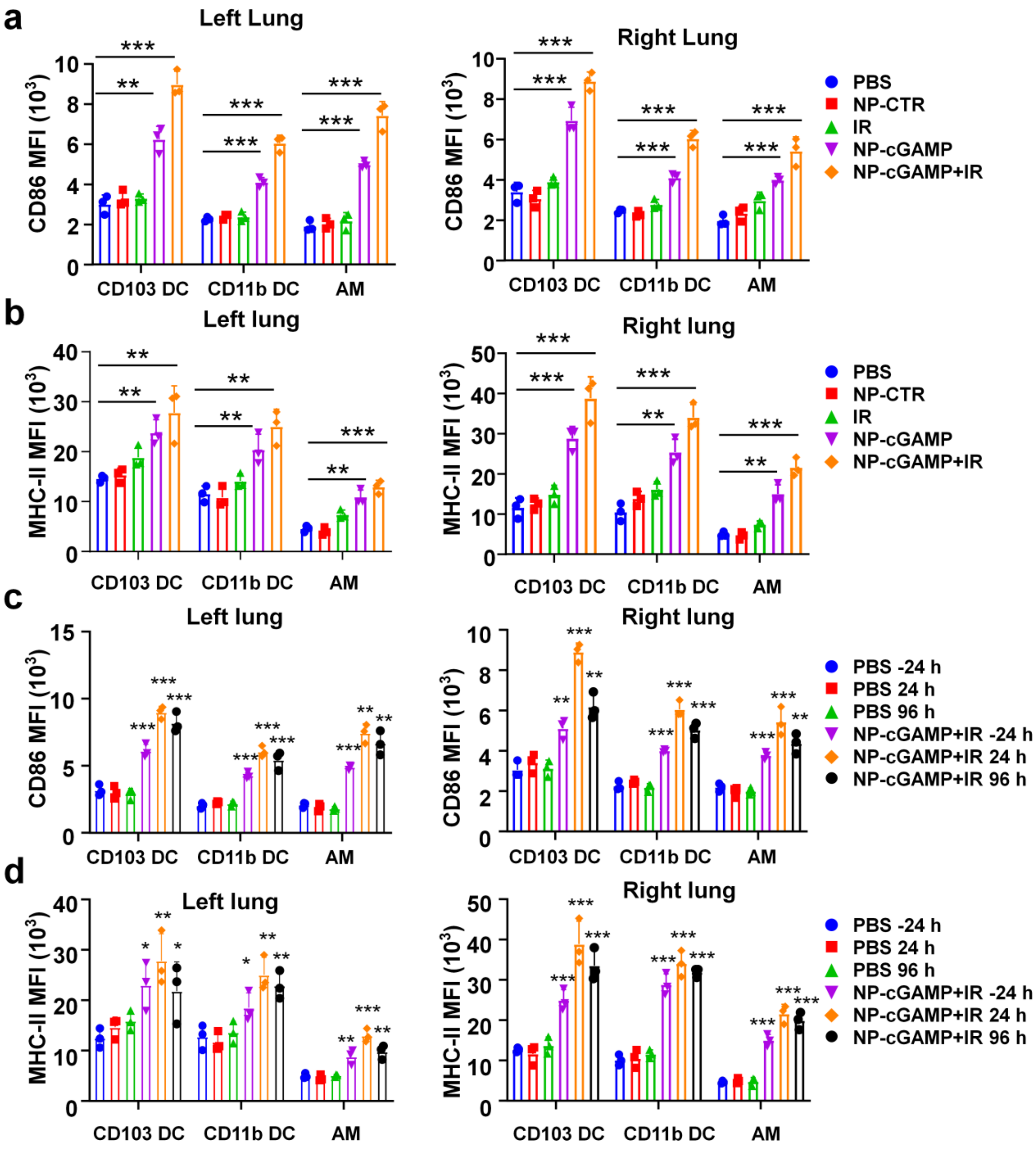

Supplementary Fig. 11. Inhalation of NP-cGAMP plus IR activates APCs in 4T1-luc lung metastases. a,b, 24h after the last inhalation, the mice under indicated treatment were sacrificed and both metastases-bearing lungs were dissected for FACS analysis. Significantly increased expressions of CD86 molecule and MHC-II on APCs were observed in both lungs of the mice with inhalation of NP-cGAMP with/without IR treatment. **c,d,** APC

maturation dynamics was also investigated during (-24h, 24h before the last inhalation) and 24h and 96h after the last inhalation. Data shown as mean ±SD of n=3 biologically independent mice/group. * $p<0.05$; ** $p<0.01$; *** $p<0.001$ by Student's T-test. Source data are provided as a Source Data file.

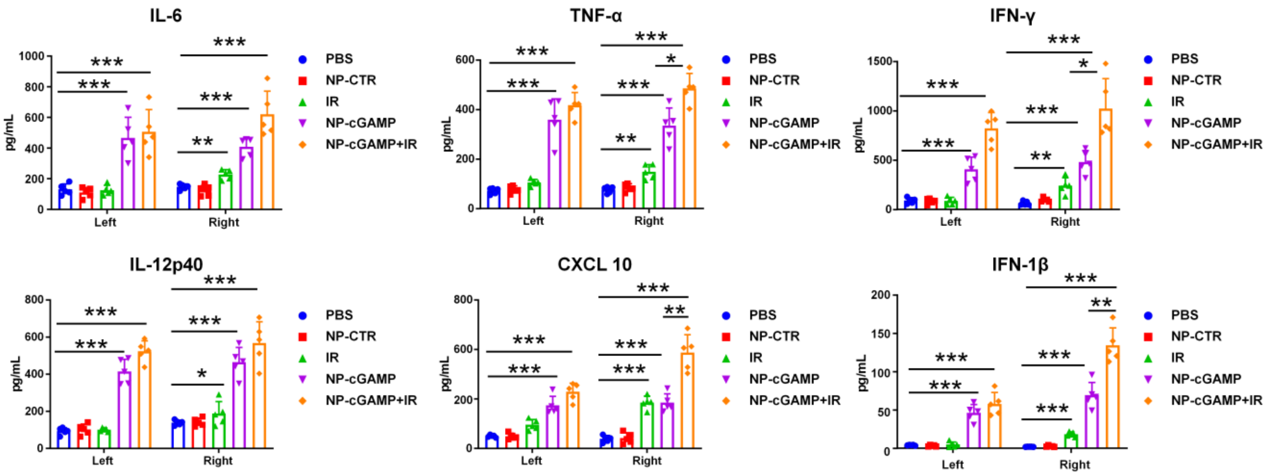

Supplementary Fig. 12. NP-cGAMP inhalation stimulates proinflammatory response in lung metastases. 24h after the last inhalation, the mice under indicated treatment were sacrificed and both the irradiated and non-irradiated metastases-bearing lungs were dissected and analyzed by ELISA. Type I IFN, IFN-1β and other proinflammatory cytokines, TNFα, IL-6, IL-12p40 and IFNγ, as well as the chemokine, CXCL10 were found to be significantly higher in both lungs of the mice with inhalation of NP-cGAMP with/without IR treatment. Data shown as mean ±SD of 5 biologically independent mice/group. * $p<0.05$; ** $p<0.01$; *** $p<0.001$ by Student's T-test. Source data are provided as a Source Data file.

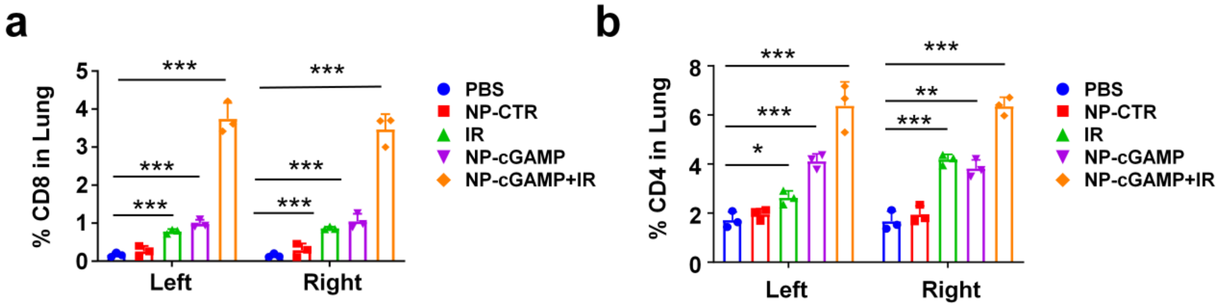

Supplementary Figure 13. NP-cGAMP inhalation plus IR promotes tumor-infiltrating lymphocytes. 24h after the last inhalation, the 4T1-luc lung metastases mice under indicated treatment were sacrificed and both metastases-bearing lungs were dissected for FACS analysis. **a** and **b**) CD8+ and CD4+ TILs increased significantly in response to inhalation of NP-cGAMP alone or in combination with IR. Data shown as mean ±SD of n=3 biologically independent mice/group. * $p<0.05$; ** $p<0.01$; *** $p<0.001$ by Student's T-test. Source data are provided as a Source Data file.

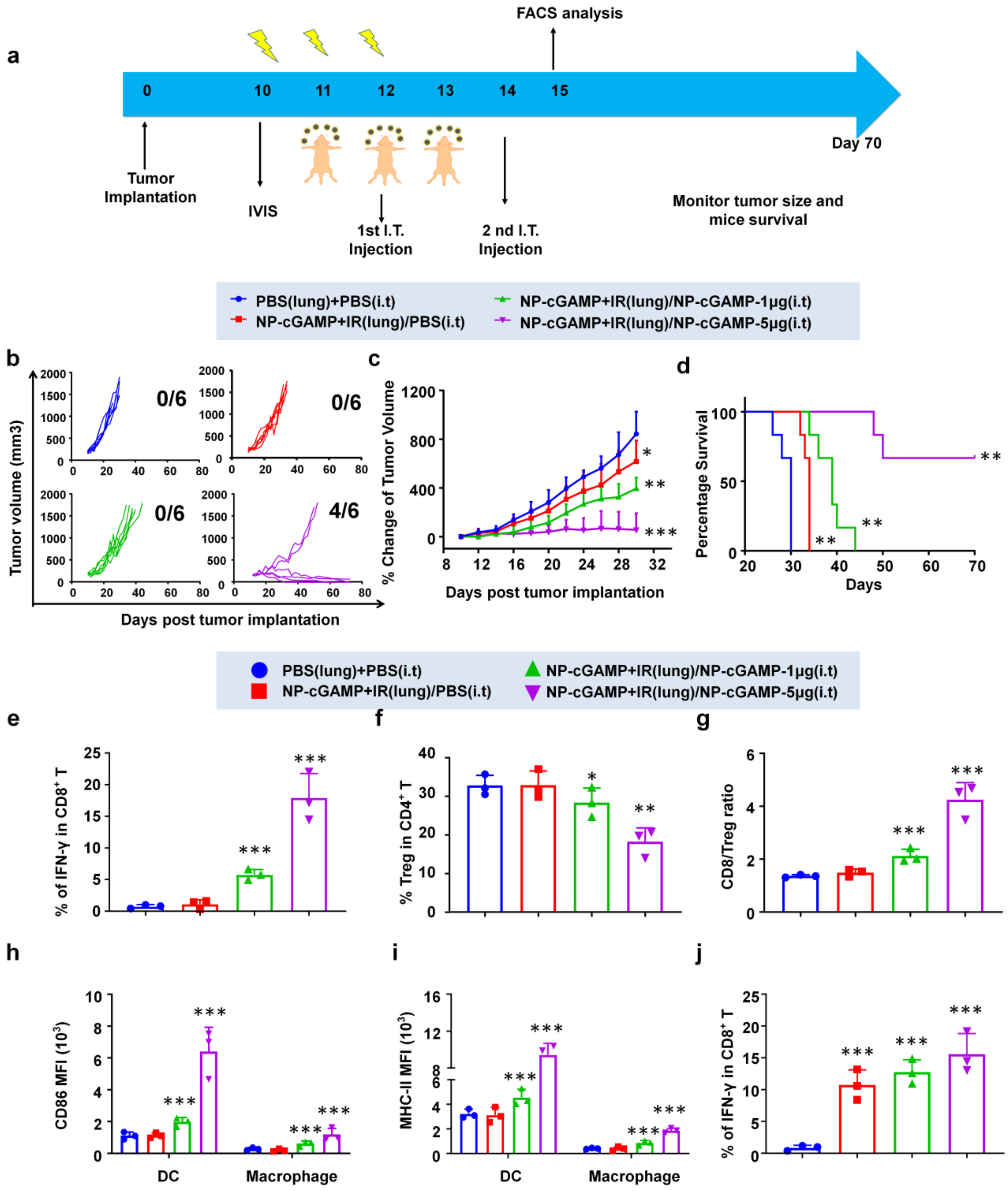

Supplementary Fig. 14. Inhalation of NP-cGAMP plus IR induces systemic anticancer immunity. In a subset of the 4T1 model (n = 6/group), the primary tumor was retained without surgical resection. **a**, As in the previous treatment study, the mice bearing both primary tumor and lung metastases were treated with inhalation

of NP-cGAMP alone or in combination with IR to the right lung. In addition to the lung treatment, the primary tumor was treated with/without intratumoral injection of NP-cGAMP (1µg or 5µg cGAMP) on day 12 and 14. **b,** Individual tumor growth up to 70 days, % change of tumor volume in each group (**c**), and survival of the mice (**d**). **e-i**, FACS analysis of the primary tumor at day 15 revealed that the lung treatment plus intratumoral treatment induced significant increase in MHC-II and CD86 expression on APCs, activated CD8+TILs and the ratio of CD8+T/Treg. **j**, There was a significant increase in activated CD8+T cells in spleens of the mice treated with the combination lung treatment with/without intratumoral NP-cGAMP. FACS data shown as mean ±SD of 3 biologically independent mice/group. * $p<0.05$; ** $p<0.01$; *** $p<0.001$ by Student's T-test. Source data are provided as a Source Data file.

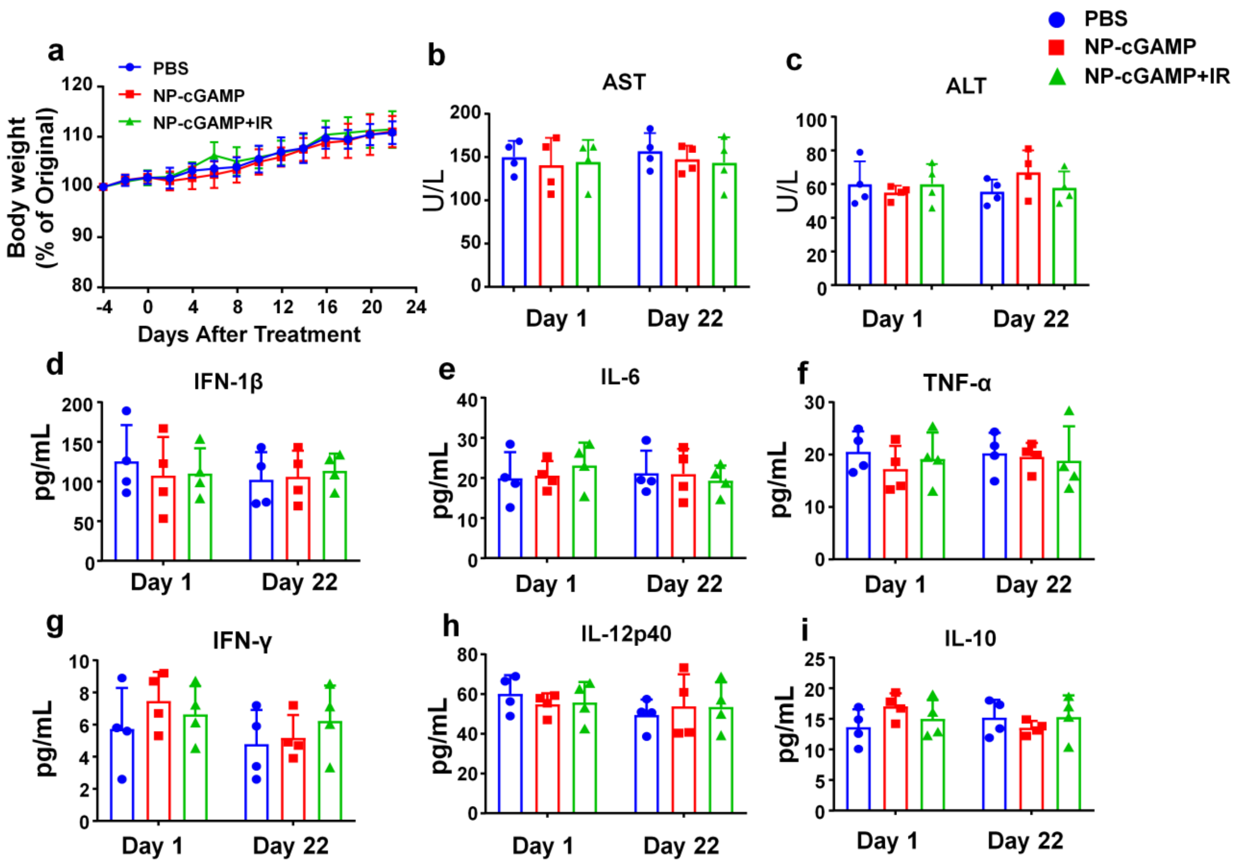

Supplementary Fig. 15. Monitoring of changes in body weight, liver function and inflammatory cytokines. a, Body weight changes were followed for 3 wks after the healthy mice were treated with inhaled NP-cGAMP alone or in combination with IR. **b,c,** Serum measurements of liver enzymes AST and ALT at day 1 and 22. **d-i,** Blood levels of various cytokines were measured at day 1 and 22. There was no significant change. Data shown as mean ±SD of n=4 biologically independent samples. Source data are provided as a Source Data file.

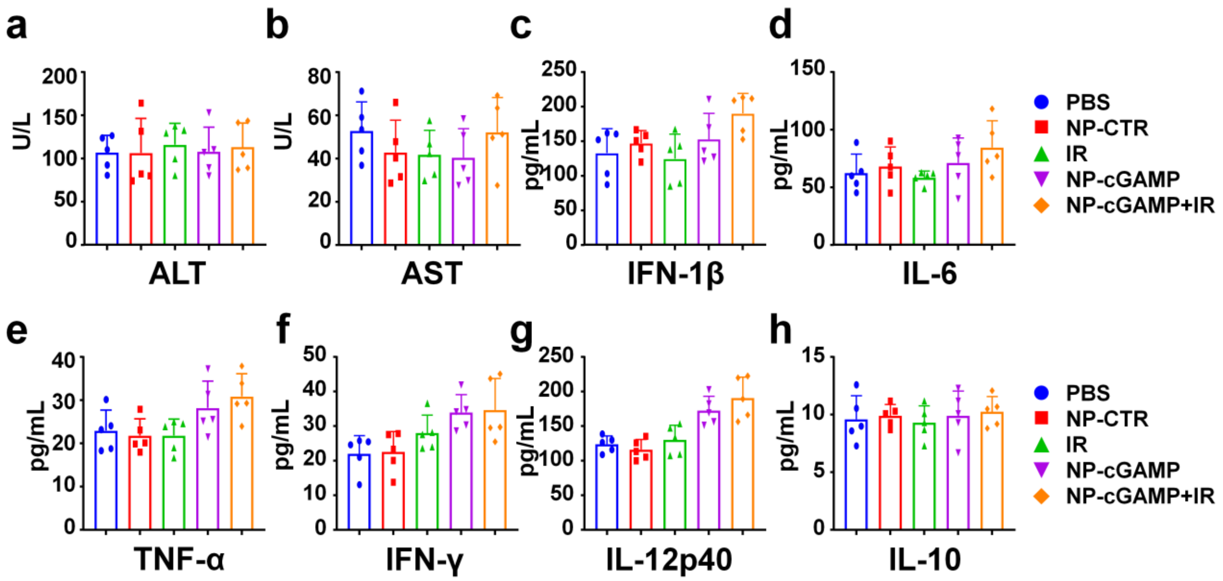

Supplementary Fig. 16. Liver function and blood cytokine levels in lung metastases-bearing mice. 24h after the indicated treatment, blood AST and ALT enzyme were measured (**a, b).** ELISA assay detected a slight increase in serum IFN-β, IFNγ, IL-6, IL-12p40 and TNFα in the NP-cGAMP+IR treatment (**b**-**h**). mean ±SD of n=5 biologically independent mice/group.

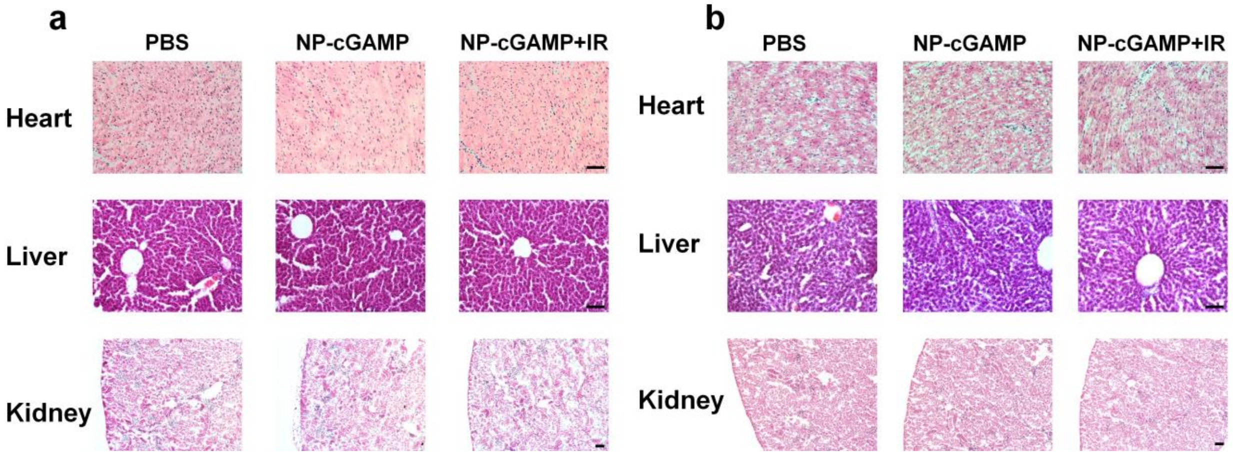

Supplementary Fig. 17. Histopathological examinations of heart, liver and kidney. a, The organs of healthy mice treated with inhalation of NP-cGAMP alone or in combination with IR were examined by H&E staining at day 22; **b,** For the lung metastases-bearing mice, the organs were examined at 24h after treatment. Representative images of n=3 biologically independent mice/group. Scale bar = 100 μm.

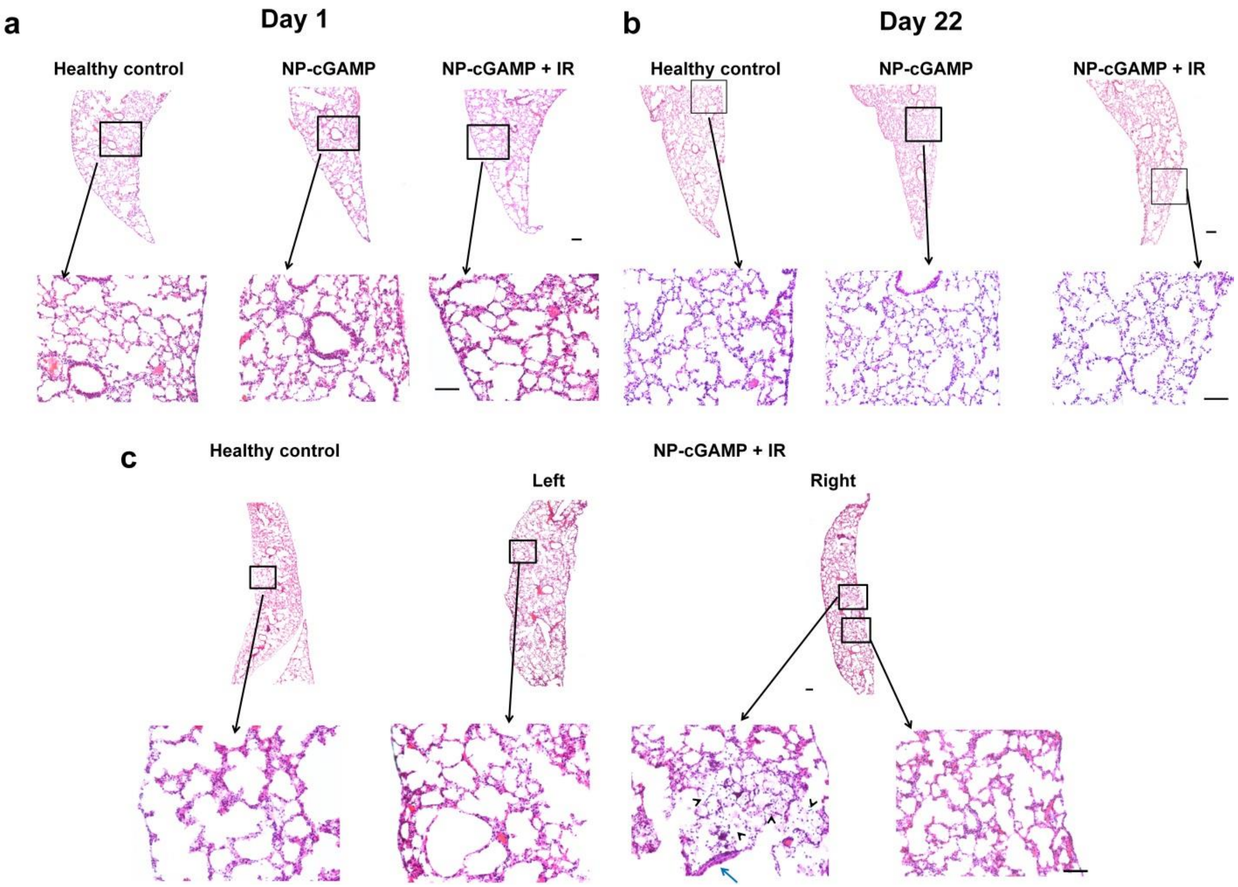

Supplementary Fig. 18. Investigation of potential adverse effects on lungs. Healthy mice (n=4/group) were treated with inhaled NP-cGAMP alone or in combination with IR to the right lung. At day 1 (**a**) and 22 (**b**), H&E staining of the right lung from indicated treatment. H&E staining showed no significant morphological change, e.g., hemorrhage, after either treatment on day 1, despite some increased infiltration of cells, likely leukocytes, observed in the lung treated with IR plus inhalation. However, on day 22, there was no sign of inflammation in the lung. Representative images of n=3 biologically independent mice/group. **c,** The 4 mice that were cured with the combination treatment were sacrificed on day 150. Representative H&E staining of the left and right (IR) lung showed no residual metastatic tumor**.** The shape of alveolar sacs, alveolar wall thickness and microvasculature in the non-irradiated left lung looked similar to the age-matched healthy counterpart. While a small area in the irradiated lung was seen to contain infiltration of macrophage-like cells (arrowheads), and slightly thickened alveolar wall (arrow), there was no other distinct morphological change. Scale bar = 200 μm for low magnification images, and scale bar = 100 μm for high magnification images.

Supplementary Table 1. Primers list for real-time quantitative PCR

Gene	Forward (5'-3')	Reverse (5'-3')
Gapdh	AAGGTCATCCCAGAGCTGAA	CTGCTTCACCACCTTCTTGA
Ifnb1	CAGCTCCAAGAAAGGACGAAC	GGCAGTGTAACTCTTCTGCAT
Cxcl9	TGGGCATCATCTTCCTGGAG	CCGGATCTAGGCAGGTTTGA
Cxcl10	CCTCATCCTGCTGGGTCTG	CTCAACACGTGGGCAGGA
Tnf	CCAGTCTGTATCCTTCTAA	TTGTGTTTCTGAGTAGTTG
Il6	TGATGCACTTGCAGAAAACA	ACCAGAGGAAATTTTCAATAGGC
Il12b	GATGACATGGTGAAGACGGC	AGGCACAGGGTCATCATCAA
Il1b	GAAAGACGGCACACCCACC	AGACAAACCGCTTTTCCATCTTC
Ifna1	AAGGACAGGAAGGATTTTGGATT	GAGCCTTCTGGATCTGTTGGTT
Il10	ATAACTGCACCCACTTCCCA	GGGCATCACTTCTACCAGGT
Tgfb1	CACCGGAGAGCCCTGGATA	TGTACAGCTGCCGCACACA

Supplementary Table 2. Antibodies list for flow cytometry

Antibody	Clone	Fluorophore
CD3	17A2	FITC
CD4	GK1.5	PE
CD8α	53-6.7	PerCP-Cyanine5.5
FOXP-3	MF-14	Alexa Fluor® 647
CD45	30-F11	APC-Cyanine7
CD45	30-F11	Pacific Blue
CD11b	M1/70	FITC
CD11b	M1/70	PE-Cyanine7
Ly-6G/Ly-6C (Gr-1)	RB6-8C5	APC
F4/80	BM8	PE
CD11c	N418	FITC
CD86	GL-1	APC-Cyanine7
I-A/I-E (MHC-II)	M5/114.15.2	Brilliant Violet 510™
I-A/I-E (MHC-II)	M5/114.15.2	APC
IFN-γ	XMG1.2	Brilliant Violet 510™
H-2K^b SIINFEKL Tetramer	D1.16	APC
CD103	2E7	Pacific Blue
CD16/CD32 (Fc Block)	93	N.A

*All the antibodies are purchased from Biolegend (San Diego, CA) except anti mouse FOXP-3-Alexa Fluor® 647 is from eBioscience (Thermo Fisher Scientific, Waltham, MA). H-2K^b SIINFEKL Tetramer-APC is provided by NIH Tetramer Core Facility (Emory University, Atlanta, GA).
