# Supplementary material for: An inhalable nanoparticulate STING agonist synergizes with radiotherapy to confer long-term control of lung metastases
Source: Nat Commun. 2019 Nov 8;10:5108. doi: 10.1038/s41467-019-13094-5 (PMC6841721; doi:10.1038/s41467-019-13094-5)
Supplement: Supplementary file 3 — Reporting Summary [file 41467_2019_13094_MOESM3_ESM.pdf]

nature research

Corresponding author(s): Dawen Zhao

Reporting Summary

Nature Research wishes to improve the reproducibility of the work that we publish. This form provides structure for consistency and transparency in reporting. For further information on Nature Research policies, see Authors & Referees and the Editorial Policy Checklist.

Statistical parameters

When statistical analyses are reported, confirm that the following items are present in the relevant location (e.g. figure legend, table legend, main text, or Methods section).

n/a	Confirmed	
☐	☒	The exact sample size (n) for each experimental group/condition, given as a discrete number and unit of measurement
☐	☒	An indication of whether measurements were taken from distinct samples or whether the same sample was measured repeatedly
☐	☒	The statistical test(s) used AND whether they are one- or two-sided<br>*Only common tests should be described solely by name; describe more complex techniques in the Methods section.*
☒	☐	A description of all covariates tested
☒	☐	A description of any assumptions or corrections, such as tests of normality and adjustment for multiple comparisons
☐	☒	A full description of the statistics including central tendency (e.g. means) or other basic estimates (e.g. regression coefficient) AND variation (e.g. standard deviation) or associated estimates of uncertainty (e.g. confidence intervals)
☒	☐	For null hypothesis testing, the test statistic (e.g. F, t, r) with confidence intervals, effect sizes, degrees of freedom and P value noted<br>*Give P values as exact values whenever suitable.*
☒	☐	For Bayesian analysis, information on the choice of priors and Markov chain Monte Carlo settings
☒	☐	For hierarchical and complex designs, identification of the appropriate level for tests and full reporting of outcomes
☒	☐	Estimates of effect sizes (e.g. Cohen's d, Pearson's r), indicating how they were calculated
☐	☒	Clearly defined error bars<br>*State explicitly what error bars represent (e.g. SD, SE, CI)*

Our web collection on statistics for biologists may be useful.

Software and code

Policy information about availability of computer code

Data collection	Bruker Paravision 6.0, NIS Element AR, Living Image
Data analysis	MATLAB, GraphPad, NIS Elements AR, Living Image, FlowJo, and Excel

For manuscripts utilizing custom algorithms or software that are central to the research but not yet described in published literature, software must be made available to editors/reviewers upon request. We strongly encourage code deposition in a community repository (e.g. GitHub). See the Nature Research guidelines for submitting code & software for further information.

Data

Policy information about availability of data

All manuscripts must include a data availability statement. This statement should provide the following information, where applicable:

- Accession codes, unique identifiers, or web links for publicly available datasets
- A list of figures that have associated raw data
- A description of any restrictions on data availability

The data that support the findings of this study are available from the corresponding author upon reasonable request. Raw data are provided for Figure1, 2, 3, 4, 6, 7, 8, and Supplementary Figure 1, 2, 3, 4, 5, 9, 11, 12, 13, 14, 15, 16.

Field-specific reporting

Please select the best fit for your research. If you are not sure, read the appropriate sections before making your selection.

☒ Life sciences ☐ Behavioural & social sciences ☐ Ecological, evolutionary & environmental sciences

For a reference copy of the document with all sections, see nature.com/authors/policies/ReportingSummary-flat.pdf

Life sciences study design

All studies must disclose on these points even when the disclosure is negative.

Sample size	No prior sample-size calculation was performed. In all experimental treatment studies, a sample size of 6-8 was used initially. Statistical analysis was performed on the collected data and the sample size was determined to be sufficient based on the size and consistency of the differences between groups.
Data exclusions	Exclusion criteria were not used in this study, as such no data were excluded.
Replication	All chemicals and cell lines were purchased from the vendors with sufficient quality controls. All experiments were recorded in a repeatable manner and were found to be highly reproducible.
Randomization	All cells and animals were randomized among groups.
Blinding	The imager of MRI was blinded to the group allocation. Otherwise, the data analyses on luminescense intensity, genome editing efficiency, etc. were directly calculated with corresponding software, without decision making by the researchers.

Reporting for specific materials, systems and methods

Materials & experimental systems

n/a	Involved in the study
☐	☒ Unique biological materials
☐	☒ Antibodies
☐	☒ Eukaryotic cell lines
☒	☐ Palaeontology
☐	☒ Animals and other organisms
☒	☐ Human research participants

Methods

n/a	Involved in the study
☒	☐ ChIP-seq
☐	☒ Flow cytometry
☒	☐ MRI-based neuroimaging

Unique biological materials

Policy information about availability of materials

Obtaining unique materials	PS coated NPs carrying cGAMP are synthesized in the lab. The NPs are available from the corresponding author upon reasonable request.

Antibodies

Antibodies used	Antibodies used in the current study have been listed as Table S2.
Validation	All the antibodies have validation statements on the vendors website as https://www.biolegend.com, https://www.thermofisher.com

Eukaryotic cell lines

Policy information about cell lines

Cell line source(s)	4T1-Luc breast cancer cells were from Dr. David R. Soto-Pantoja (Wake Forest University), B16-OVA cells were from Dr. Yong Lu (Co-author), and bEND.3 cells were from ATCC

Authentication	None of the cells were authenticated in the lab.
Mycoplasma contamination	The cells were routinely tested negatively for mycoplasma.
Commonly misidentified lines (See ICLAC register)	No commonly misidentified cell lines were used.

Animals and other organisms

Policy information about studies involving animals; ARRIVE guidelines recommended for reporting animal research

Laboratory animals	C57BL/6 mice (6–8 weeks, female:male at 1:1) were purchased from Charles River Laboratories. BALB/c mice (8-10 weeks,female) were purchased from Charles River Laboratories
Wild animals	The study did not involve wild animals.
Field-collected samples	The study did not involve samples collected from the field.

Flow Cytometry

Plots

Confirm that:

☒ The axis labels state the marker and fluorochrome used (e.g. CD4-FITC).

☒ The axis scales are clearly visible. Include numbers along axes only for bottom left plot of group (a 'group' is an analysis of identical markers).

☒ All plots are contour plots with outliers or pseudocolor plots.

☒ A numerical value for number of cells or percentage (with statistics) is provided.

Methodology

Sample preparation	All cells were cultured according to the standard protocols from the distributors. Doublets and debris of dead cells were excluded before various gating strategies were applied.
Instrument	BD Canto II flow cytometer
Software	FlowJo software
Cell population abundance	20,000 to 30,000 events were collected in predetermined gates for each group
Gating strategy	Gates and quadrants were set based on isotype control staining.

☒ Tick this box to confirm that a figure exemplifying the gating strategy is provided in the Supplementary Information.
